# Investigation of Shape Transformations of Vesicles, Induced by Their Adhesion to Flat Substrates Characterized by Different Adhesion Strength

**DOI:** 10.3390/ijms222413406

**Published:** 2021-12-14

**Authors:** Jeel Raval, Aleš Iglič, Wojciech Góźdź

**Affiliations:** 1 Institute of Physical Chemistry, Polish Academy of Sciences, Kasprzaka 44/52, 01-224 Warsaw, Poland; jraval@ichf.edu.pl; 2 Faculty of Electrical Engineering, University of Ljubljana, Tržaška 25, 1000 Ljubljana, Slovenia; Ales.Iglic@fe.uni-lj.si

**Keywords:** lipid vesicles, bending energy, vesicle adhesion, vesicle shape, membrane budding, shape transitions

## Abstract

The adhesion of lipid vesicles to a rigid flat surface is investigated. We examine the influence of the membrane spontaneous curvature, adhesion strength, and the reduced volume on the stability and shape transformations of adhered vesicles. The minimal strength of the adhesion necessary to stabilize the shapes of adhered vesicles belonging to different shape classes is determined. It is shown that the budding of an adhered vesicle may be induced by the change of the adhesion strength. The importance of the free vesicle shape for its susceptibility to adhesion is discussed.

## 1. Introduction

The adhesion of biological cells and vesicles is ubiquitous in nature. It occurs during endo- and exocytosis, when cells communicate with their environment. The adhesion of cells plays a crucial role in tissue morphogenesis, migration, self-recognition, immune response, synapse formation and embryogenesis. Adhesion may play an important role in drug delivery by liposomes when they attach to the plasma membrane to release their content into the target sites. It is also important in biotechnological applications such as material implantation or biosensors when the membrane binds to a substrate. There are many different mechanisms of the adhesion. They may result from attraction of the bilayer to other bilayers [1,2,3] or to a substrate [4,5,6,7,8,9,10,11]. The size of the surface area of a vesicle, which is in contact due to adhesion, may depend on the strength of the interactions or the concentration of the sticker molecules [12,13]. It may also depend on the external force, which brings two vesicles or a vesicle and a solid substrate into contact. In this study, we focus on the shape transformations of lipid vesicles caused by their adhesion to a flat and rigid substrate. We study the stability of adhered vesicles for different values of reduced adhesion strength of an underlying flat solid substrate. We determine the minimal strength of the adhesion, which allows for stabilization of the adhered vesicles of different classes.

The ensemble that mimics the experimental situation that we investigate is the one with fixed topology, constant surface area *S*, and volume *V*. Such a physical situation is well described by the elastic energy [14,15,16] given by:

(1) $$F_{b}=\frac{\kappa }{2}\int_{S}dS{\left(C_{1}+C_{2}-C_{0}\right)}^{2}-WA$$

where κ is the bending rigidity, $C_{1}$ and $C_{2}$ are the principal curvatures, $C_{0}$ is the spontaneous curvature, *W* is the adhesion strength, *A* the adhesion area and the integral (1) is taken over the surface of a closed vesicle. No topology changes are assumed, therefore the integral over the Gaussian curvature contributes a constant value and is omitted in Equation (1). When the spontaneous curvature is imposed by different densities of the solution inside and outside of a vesicle [17], the effect of gravity can be important. However, the spontaneous curvature can be imposed by a variety of mechanisms, which do not result in the density difference. We are investigating only such systems.

Vesicle shapes can be well approximated in numerical calculations by surfaces that are rotationally symmetric. The numerical calculations can be performed when the shape of a vesicle is parameterized with the angle between the rotation axis and the line tangent to the shape profile, θ(s), as a function of the arclength s. The shape profile in Cartesian coordinates is calculated from parametric equations (r(s),z(s)), where the radius r(s) and the height z(s) at a given value of a parameter *s* are calculated from θ(s) according to:

(2) $$r\left(s\right)=\int_{0}^{s}ds^{\prime }\cos\left(\theta \left(s^{\prime }\right)\right),$$

(3) $$z\left(s\right)=\int_{0}^{s}ds^{\prime }\sin\left(\theta \left(s^{\prime }\right)\right).$$

In order to parameterize a closed shape, the following constraints must be satisfied:

(4) θ(0)=0,

(5) $$\theta (L_{s})=\pi ,$$

(6) $$r(L_{s})\geq 0,$$

where $L_{s}$ is the length of the shape profile. The Equations (4) and (5) guarantee that the profile is smooth at the ends and Equation (6) accounts for the fact that the vesicle may touch the substrate at a distance $r(L_{s})$ from the axis of rotation.

The functional (1) with the shape profile parametrized by θ(s) is given by:

(7) $$F=\frac{\kappa }{2}\left[\pi r{\left(L_{s}\right)}^{2}C_{0}^{2}+\left(2\pi \right)\int_{0}^{L_{s}}dsr\left(s\right){\left(\frac{d\theta (s)}{ds}+\frac{\sin(\theta (s))}{r(s)}-C_{0}\right)}^{2}\right]-W\pi r{\left(L_{s}\right)}^{2},$$

where *W* is the adhesion strength and $\pi r{\left(L_{s}\right)}^{2}$ is the contact surface area. Adding explicitly the adhesion strength *W* to the functional (7) allows us to compare the stability of different solutions for adhered vesicles. The calculations are performed for fixed adhesion strength *W*, and the adhesion radius is determined during the minimization process. In our previous study [7], the calculations were performed with fixed adhesion radius and the adhesion strength *W* was not specified. The functional (7) is minimized numerically. The function describing the shape profile θ(s) is approximated by the Fourier series,

(8) $$\theta \left(s\right)=\theta_{0}\frac{s}{L_{s}}+\sum_{i=1}^{N}a_{i}\sin\left(\frac{\pi }{L_{s}}i\cdot s\right),$$

where *N* is the number of Fourier modes, and $a_{i}$ are the Fourier amplitudes. We use 80 independent Fourier modes in the numerical calculations. Large number of the amplitudes, of the order of one hundred, is required in order to accurately parameterize complex shapes. $\theta_{0}$ is the angle at the point where the membrane touches the substrate, $\theta_{0}=\theta \left(L_{s}\right)$. We can define this angle as the contact angle and assume that it is $\theta_{0}=\pi$ in order to keep the profile of the vesicle smooth at all points. The value $R=r(L_{s})$ defines the contact area, *A*, given by $A=\pi R^{2}$, which appears in Equation (7). Thus, we can define $R_{adh}=R$ as an adhesion radius. The functional variation is replaced by the minimization of the function of many variables [18]. The function (7) is minimized with respect to the amplitudes $a_{i}$ and the length of the shape profile $L_{s}$, under the constraints of constant surface area, *S* volume *V*, and adhesion radius *R*, where

(9) $$S=\pi r{\left(L_{s}\right)}^{2}+2\pi \int_{0}^{L_{s}}dsr\left(s\right),$$

(10) $$V=\pi \int_{0}^{L_{s}}dsr^{2}\left(s\right)\sin\theta (s),$$

(11) $$R=\int_{0}^{L_{s}}ds^{\prime }\cos\left(\theta \left(s^{\prime }\right)\right).$$

The volume, $V_{0}$, and the radius, $R_{0}$, of the sphere having the same surface area, *S*, as the investigated vesicle are chosen as the volume and length units, respectively [5,19]:

(12) $$R_{0}=\sqrt{S/4\pi }$$

(13) $$V_{0}=\frac{4}{3}\pi R_{0}^{3}$$

The reduced volume is defined as $v=V/V_{0}$, the reduced adhesion strength as $w=WR_{0}^{2}/\kappa$, the reduced spontaneous curvature $c_{0}=C_{0}R_{0}$, the reduced free energy f=F/8πκ, and the reduced adhesion radius $r_{adh}=R_{adh}/R_{0}$.

## 2. Results

The stability of vesicles adhered to flat substrates characterized by different values of the reduced adhesion strength *w* is investigated. The values of the adhesion strength depends on the material used to build the substrate or the composition of the vesicle’s membrane. The adhesion strength may be also varied by the change of the thermodynamic parameters [11]. We have studied the shapes of adhered vesicles characterized by a few values of the reduced volume *v* and the reduced spontaneous curvature $c_{0}$. We have determined the minimal reduced adhesion strength *w* for which adhered vesicles become more stable than a free vesicle. The range of the stability of different classes of vesicle shapes for different reduced adhesion strength *w* was examined. Possible shape transitions between adhered vesicles are also investigated.

**Figure 1:**
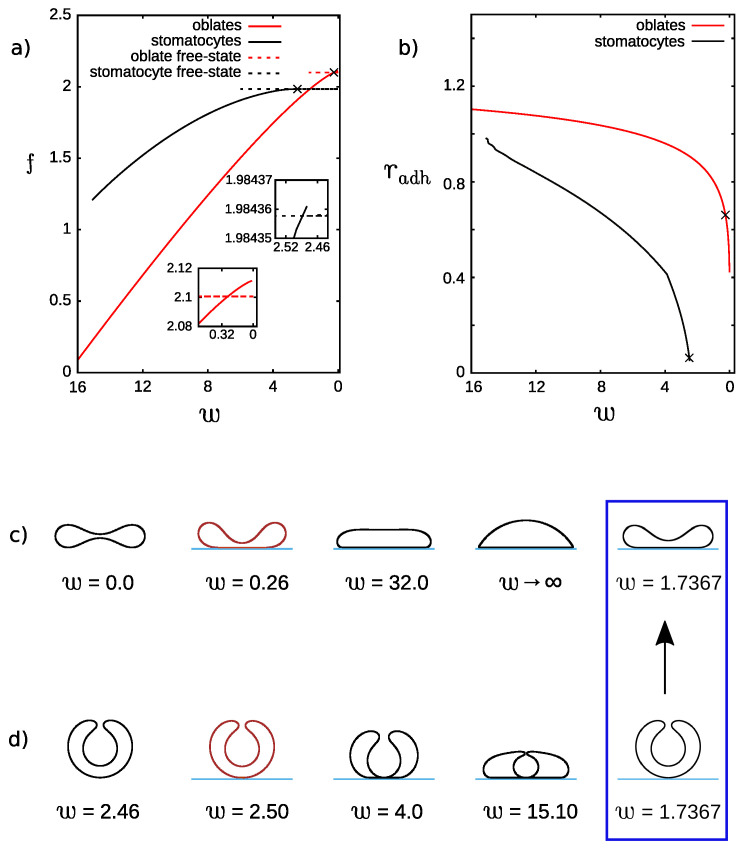
(**a**) The dependence of the reduced free energy, *f*, on the reduced adhesion strength, *w*, and (**b**) the dependence of the reduced adhesion radius, $r_{adh}$ on the reduced adhesion strength, *w*, for the reduced volume v=0.545 and reduced spontaneous curvature $c_{0}=0.0$. The crosses denote the points where the stable adhered vesicles for the smallest values of the adhesion strength *w* are formed. These points are obtained from intersection of the reduced free energy curves visualized in larger scale in the insets. Shape profiles for stomatocytes and oblate vesicles obtained for the following sets of the parameters: (**c**) adhesion strength w=0.0,0.26,32.0,w→∞,1.7367, adhesion radius $r_{adh}=0.0,\;0.6611,\;1.1596,\;1.2999,\;0.8656$. (**d**) adhesion strength w=2.46,2.50,4.0,15.10 (limiting shape),1.7367, adhesion radius $r_{adh}=0.0,\;0.0636,\;0.4229,\;0.9827,\;0.0$. The shapes at the intersection of dashed black (free stomatocyte) and solid red (adhered oblate) curves are shown in the blue frame.

The vesicles with zero membrane spontaneous curvature are investigated first. Compared to former studies [5] of adhesion of vesicles with the spontaneous curvature $c_{0}=0$, we keep the reduced volume of the vesicle fixed. Without adhesion, for low reduced volume and zero spontaneous curvature, the stable vesicles with $c_{0}=0$ are stomatocytes. Free stomatocytes become unstable for larger values of the reduced spontaneous curvature and the reduced volume. For the reduced volume v=0.545, it is possible to obtain three different solutions for free vesicles: stomatocyte, oblate, and prolate. Multiple solutions can be obtained not only for v=0.545, but also for a wide range of the reduced volume. We have investigated how the stability of stomatocytes and oblate vesicles changes with the change of the adhesion strength. Stable adhered prolate vesicles were not obtained by numerical minimization when the calculations were performed for fixed reduced adhesion strength *w* and the radius of adhesion was free to change. Instead, the free vesicle was obtained for the range of the reduced adhesion strength *w* studied by us. The change of the reduced free energy *f* as a function of the reduced adhesion strength *w* is shown in Figure 1a. The change of the reduced adhesion radius $r_{adh}$ as a function of the reduced adhesion strength *w* is shown in Figure 1b. From the dependence of the reduced free energy *f* on the reduced adhesion strength *w*, we can determine when the adhered vesicle becomes more stable than the free vesicle, as shown in insets of Figure 1a.

The evolution of the shapes for adhered oblate vesicles and stomatocytes with the change of the reduced adhesion strength *w* is presented in Figure 1c,d, respectively. In the first column, the vesicles in a free state are shown as a reference point to the vesicles in adhered states. In the second column, the stable adhered vesicle shapes for the oblate and stomatocyte branch are presented (stable for the lowest adhesion strength *w*). If only the states with the lowest energy are considered, it can be inferred from Figure 1a that, initially, for low adhesion strength the stable vesicles are stomatocytes that are not adhered. However, the first stable adhered vesicles are oblate ones. The energy of the free stomatocytes is marked by the black dashed horizontal line, which is below the solid red line denoting the energy of adhered oblate vesicles. These two lines intersect before the adhered stomatocytes (denoted by the solid black curve) become stable.

Thus, we may speculate that adhesion may be accompanied by a change of a vesicle shape from stomatocyte to oblate. This change of the shape takes place between the vesicles pictured in the last column of Figure 1c,d, where the configurations with the same free energy are presented. The arrow indicates the direction of a possible energetically favorable shape transition where the stomatocyte vesicle would be flattened and transformed into an adhered oblate vesicle with an increase of the adhesion strength. The limiting configurations which result from a very large adhesion strength *w* are presented in the fourth column of Figure 1c,d. In the third column, the intermediate configurations are shown.

It has to be noted that there might exist stable or metastable configurations not studied here due to the limitation of the numerical procedure. For oblate vesicles, the configuration with the adhered area in a shape of a ring may be more stable for small values of the adhesion strength *w*. However, when a significant difference of the energy between a free oblate and a free stomatocyte vesicle is considered, it is highly probable that the gain of the adhesion energy of oblate vesicle (with the center detached from the substrate) will not compensate sufficiently the loss of the bending energy to obtain a configuration with the energy lower than the energy of the adhered stomatocyte vesicle. As expected, we also notice that in the range of small *w* for oblate adhered vesicles smaller changes of the adhesion strength induce large changes of the adhesion radius than it is in the case of stomatocyte ones, as shown in Figure 1b.

**Figure 2:**
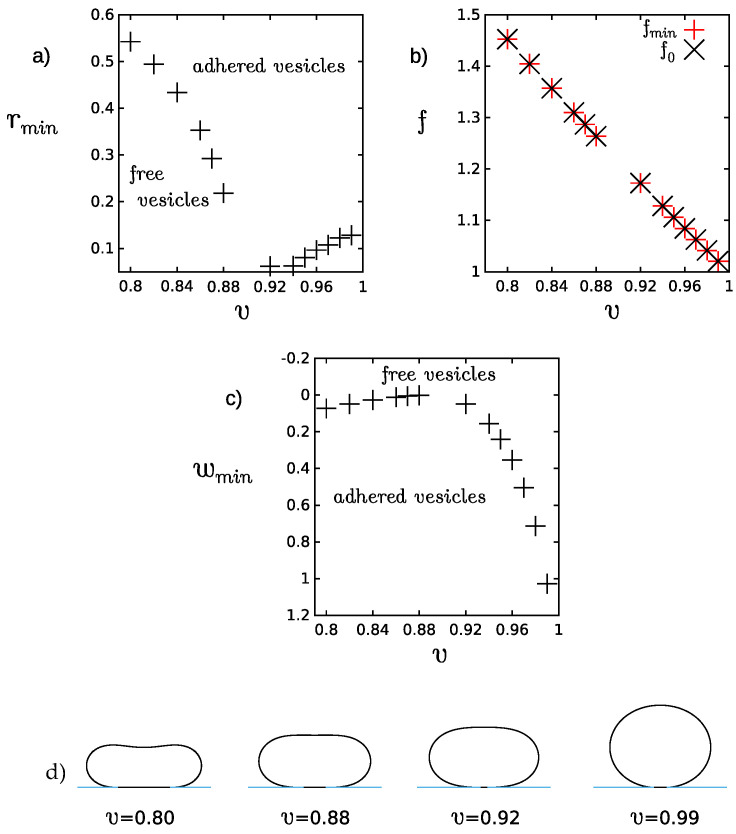
The dependence of (**a**) adhesion radius ($r_{min}$), (**b**) free energy (*f*), and (**c**) the minimal adhesion strength ($w_{min}$) on the reduced volume *v*. The spontaneous curvature is $c_{0}=0.0$. (**d**) The shape profiles represent the stable adhered vesicles for the smallest adhesion strength $w_{min}$ for different values of the reduced volume *v*.

The transition from a free state to an adhered state depends on the reduced volume of a vesicle [5]. In order to examine this dependence, oblate vesicles with several values of the reduced volume *v* have been studied. The range of the reduced volume close to the limiting spherical shape have been investigated. In Figure 2, the plots which illustrate the dependence of the radius of adhesion, $r_{adh}$, the reduced free energy, *f*, the smallest values of the adhesion strength for which the adhered vesicles become more stable than the free vesicles, $w_{min}$, as a function of the reduced volume *v* are presented. The adhesion radius corresponding to the adhesion strength $w_{min}$ is denoted as $r_{min}$. It should be noted that the radius of adhesion does not change monotonically with *v* in the range 0.80<v<0.99. For the smaller values of *v* in this range, $r_{min}$ decreases with increasing *v* and for the larger values of *v*, $r_{min}$ increases with *v*. These two tendencies can be explained by analyzing the shape profiles of the adhered vesicles shown in Figure 2. In the range of smaller *v*, the adhered vesicle has a concave shape, while in the range of larger *v*, its shape is convex. In the intermediate range about v=0.90, both free and adhered vesicles are almost flat at the top and the bottom. It can be noticed in the plot of the adhesion strength $w_{min}$ that in the flat region, the adhered vesicles can be stabilized with the smallest values of $w_{min}$. It can be attributed to the fact that in this intermediate region only small deformations of the vesicle are needed to stabilize the vesicles adhered to a flat substrate.

For smaller reduced volume, *v*, smaller values of the reduced adhesion strength, $w_{min}$, are needed to stabilize adhered vesicles than for the larger values of *v*. The smaller values of the adhesion strength $w_{min}$ can be related to the larger values of the adhesion radius. It implies that the adhesion surface is larger and thus the adhesion energy is significant even for small values of the adhesion strength $w_{min}$. Such behavior is possible for lower reduced volume *v* since in this case the vesicles have more freedom to be deformed and the increase of the elastic energy of the vesicles due to adhesion can be compensated by the gain of the adhesion energy, which depends on the radius of adhesion. This mechanism does not apply to the vesicles with larger reduced volume *v* because they have less freedom to be deformed. If v≈1.0 the loss of elastic energy is compensated by the adhesion energy in such a way that the radius of adhesion remains small and the adhesion strength is continuously increased as shown in Figure 2a,c. The shape at v=1.0 (sphere) cannot be deformed at all.

With increasing spontaneous curvature of vesicles, their shapes are more and more complex [20]. We have investigated how the complexity of a vesicle shape influences the process of adhesion. The calculations for the reduced volume v=0.545 and the reduced spontaneous curvature $c_{0}=2.4$ were performed. For these parameters we have not obtained adhered stomatocyte vesicles, but we have obtained adhered oblate vesicles and two additional types of vesicles as compared to the case with the same reduced volume and the reduced spontaneous curvature $c_{0}=0$. These two additional solutions are oblate vesicles with a bead and prolate ones.

**Figure 3:**
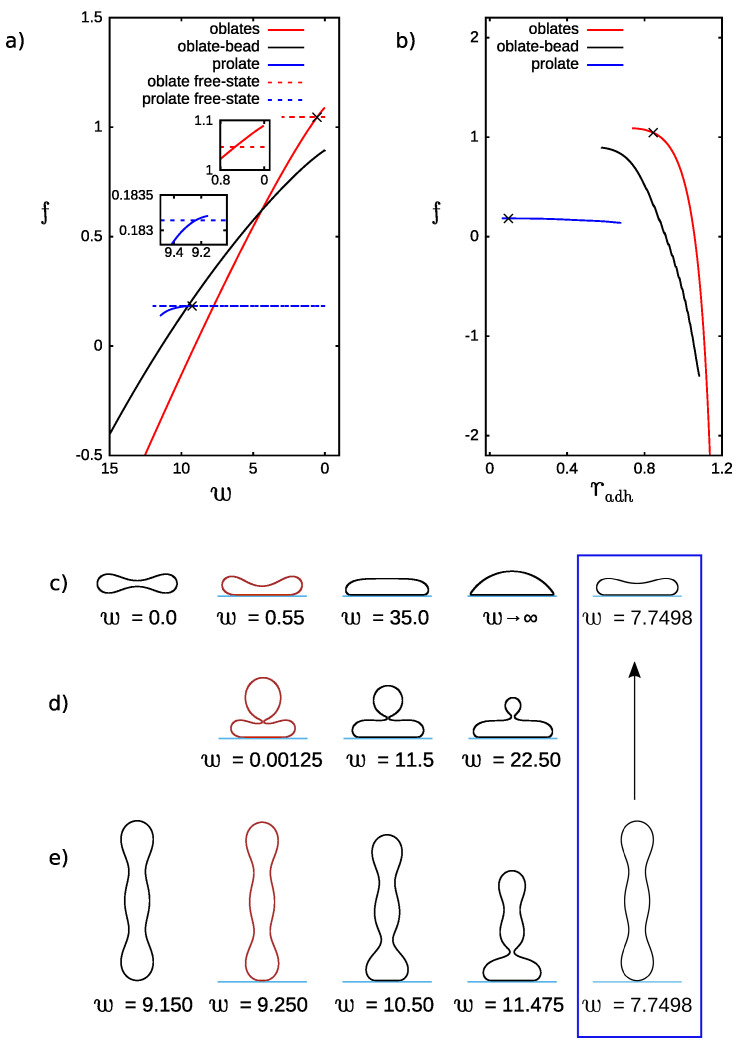
The dependence of the reduced free energy, *f*, on (**a**) the adhesion strength, *w*, and (**b**) adhesion radius, $r_{adh}$, for the reduced volume v=0.545 and spontaneous curvature $c_{0}=2.4$. The crosses denote the points where the stable adhered vesicles for the smallest values of the adhesion strength *w* are formed. These points are obtained from intersection of the reduced free energy curves visualized in larger scale in the insets. Shape profiles (**c**) adhesion strength w=0.0,0.55,35.0,w→∞ (limiting shape), 7.7498, adhesion radius $r_{adh}=0.0,\;0.8447,\;1.1666,\;1.2940,\;1.0446$. (**d**) adhesion strength w=0.00125,11.5,22.50, adhesion radius $r_{adh}=0.5769,\;0.9121,\;1.0832$. (**e**) adhesion strength w=9.150,9.250,10.50,11.475,7.7498, adhesion radius $r_{adh}=0.0,\;0.0967,\;0.3796,\;0.6791,\;0.0$. The shapes at the intersection of dashed blue (free prolate) and solid red (adhered oblate) curves are shown in the blue frame. The profiles pictured in red are the stable adhered configurations obtained for the lowest adhesion strength *w*.

The solutions with the lowest energy are either free prolate or adhered oblate vesicles. In the second column in Figure 3c,e, the first stable (with respect to the free state of the same kind) adhered oblate and prolate vesicles are shown, respectively. The energy at the transition point between stable adhered and free states of oblate and prolate vesicles is shown in the insets in Figure 3a. The oblate vesicles with a bead presented in Figure 3d exist only in an adhered state.

If only the stability of adhered states is considered, it may be noticed that for low adhesion strength, the oblate vesicles with a bead are stable, and for larger adhesion strength, oblates without a bead are stable. Sufficiently large non-zero spontaneous curvature favors free prolate shapes. Oblate shapes are favored when a vesicle adheres to a flat substrate. The adhered oblate vesicles with a bead are the result of a compromise between these two classes of shapes. When we consider the configurations of prolate vesicles attached horizontally to a flat substrate, it is highly probable that such configurations are more stable in an adhered state for small values of the adhesion strength *w*. It follows from a large difference in energy between the free prolate vesicles and the adhered oblate ones, as shown in Figure 3a. Thus, the loss of the bending energy due to the deformation of a prolate vesicle in an adhered horizontal position will be still smaller than the gain of the adhesion energy for the oblate vesicles. Unfortunately, such configurations cannot be taken into account in the current calculations since they are not axisymmetric.

**Figure 4:**
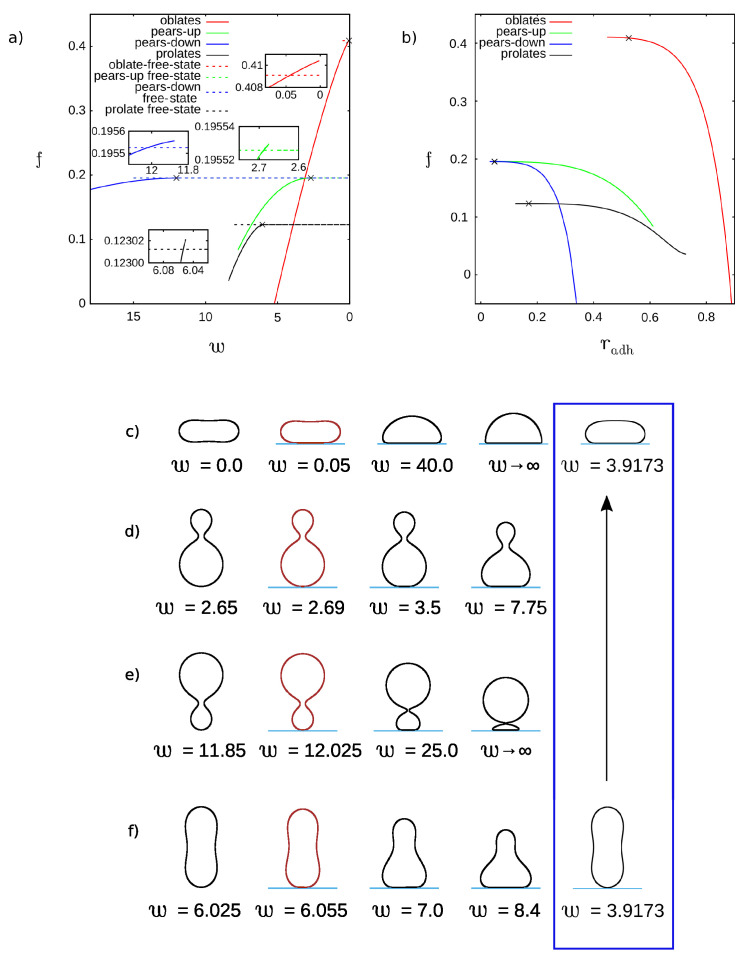
The dependence of the reduced energy, *f*, on (**a**) the reduced adhesion strength, *w*, and (**b**) reduced adhesion radius, $r_{adh}$, for the reduced volume v=0.80, and the reduced spontaneous curvature $c_{0}=2.4$. The crosses denote the points where the stable adhered vesicles for the smallest values of the adhesion strength *w* are formed. These points are obtained from intersection of the reduced free energy curves visualized in larger scale in the insets. The shape profiles are plotted in each row for following parameters: (**c**) adhesion strength w=0.0,0.05,40.0,w→∞ (limiting shape), 3.9173, adhesion radius $r_{adh}=0.0,\;0.5248,\;1.0313,\;1.1097,\;0.8531$. (**d**) adhesion strength w=2.65,2.69,3.5,7.75, adhesion radius $r_{adh}=0.0,\;0.0492,\;0.2182,\;0.6121$. (**e**) adhesion strength w=11.85,12.025,25.0,w→∞ (limiting shape), adhesion radius $r_{adh}=0.0,\;0.0478,\;0.2747,\;0.5122$. (**f**) adhesion strength w=6.025,6.055,7.0,8.4,3.9173, adhesion radius $r_{adh}=0.0,\;0.1699,\;0.5187,\;0.7276,\;0.0$. The shapes at the intersection of dashed black (free prolate) and solid red (adhered oblate) curves are shown in the blue frame.

We have also investigated the vesicles with the same non-zero reduced spontaneous curvature as in the previous case $c_{0}=2.4$, but larger reduced volume v=0.8. For this set of parameters, three different solutions are obtained for the free vesicles: prolate, pear, and oblate. Prolate and oblate vesicles have up–down symmetry. In the case of pear vesicles, we have two different states of adhered vesicles due to the lack of up-down symmetry. The first one when the smaller bead is attached to the substrate and the second one when the larger bead is attached as shown in Figure 4d,e.

The first stable adhered vesicles for oblate, pear, and prolate branches are shown in the second column of Figure 4c–f. In the first column the solutions for the vesicles in a free state are presented. In the third and forth column, the intermediate and limiting solutions for a large value of the adhesion strength *w* are shown. When we consider the solutions with the lowest energy, the free prolate vesicles are stable for lower adhesion strength *w* and adhered oblate vesicles are stable for larger *w*. In the fifth column, we present the configurations with the same free energy for free prolate and adhered oblate vesicles at the possible transformation from free to adhered vesicles for this set of parameters. This is similar to the previous case with smaller reduced volume v=0.545 and positive spontaneous curvature $c_{0}$ where the shape of the stable free state is prolate and the shape of the adhered state is oblate.

When only adhered states with the lowest energy are considered, we have a very interesting situation. The adhered oblate vesicles have the lower energy except for a small range of the adhesion strength 2.69<w<3.11 where the solution with the lower energy are adhered pear-like vesicles attached with a larger bead to the flat surface. Based on the free energy calculations as shown in Figure 4a, we may expect the existence of two transitions. At w=3.11 the energy of adhered oblate and pear-like vesicles is equal. Thus, by increasing or decreasing the adhesion strength *w*, the transition between adhered pear-like and adhered oblate vesicles can be induced. The adhered oblate vesicles are already metastable at w=0.05, but adhered pear-like vesicles are metastable only for w>2.69. However, the energy of the adhered pear-like vesicles is significantly smaller at w=2.69 than the energy of adhered oblate vesicles. Thus, we may expect that by increasing the adhesion strength the adhered oblate vesicles could be transformed to adhered pear-like vesicles.

**Figure 5:**
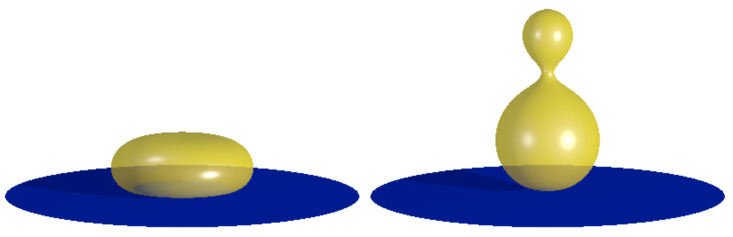
Budding of adhered vesicles induced by decreasing the adhesion strength *w*. The adhered oblate and adhered pear-like vesicles have equal energy at w=3.11 for the reduced volume v=0.80, and the reduced spontaneous curvature $c_{0}=2.4$.

Based on this result, we can speculate that in biological systems, budding may be induced by a very small variations in the adhesion strength and it can be easily reversed. It should be stressed here that no change in the distribution of the components or the spontaneous curvature is needed to induce budding. It is enough to increase the surface area of adhesion of the vesicle, for example by the change of the adhesion strength as shown in Figure 5.

Different behavior can be observed when a pear-like vesicle is attached to a substrate with its smaller spherical part or the larger spherical part. When the smaller bead is attached, the neck in the middle becomes smaller and smaller with increasing the adhesion strength. Such process may lead to budding in the end. When the larger part is attached the neck widens and the vesicle is transformed to an adhered oblate. Thus, depending on which part of the vesicle is attached to the substrate, it is possible to open or close the gate which is formed by the neck in the central part of the vesicle. In this way, by changing the radius of the neck it is possible for example to prohibit or enhance the mixing of the fluids which are contained in these two parts of the vesicles.

**Figure 6:**
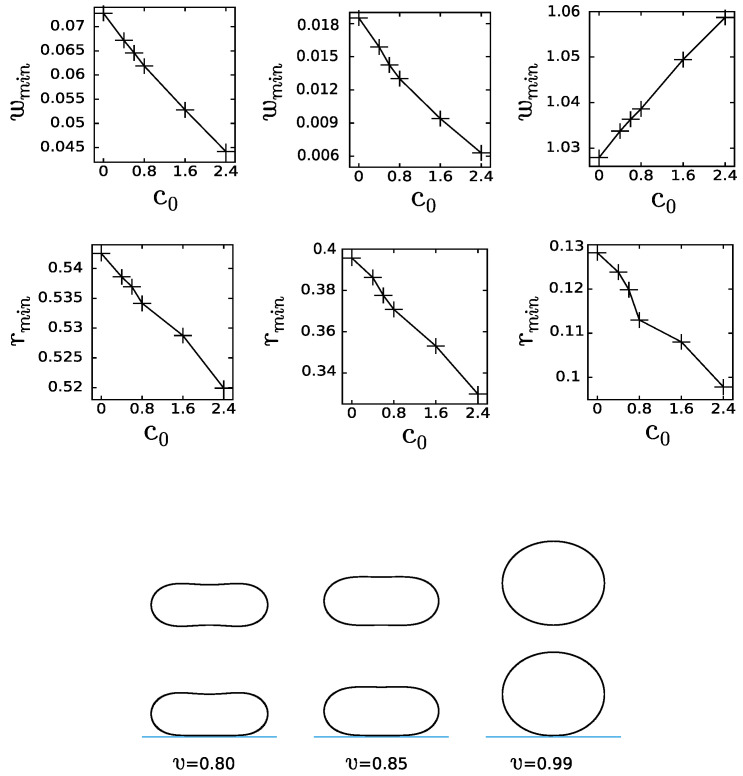
The change of the reduced adhesion radius, $r_{min}$, and the reduced adhesion strength, $w_{min}$, induced by the change of the reduced spontaneous curvature $c_{0}$ for oblate vesicles with relatively large reduced volume v=0.8,0.85,0.99. The shape profiles represent free vesicles in the first row and adhered vesicles with the reduced spontaneous curvature $c_{0}=2.4$ for the reduced volume v=0.8,0.85,0.99 in each column, respectively.

Finally, we have examined how the increase of the spontaneous curvature would influence the adhesion of oblate vesicles with relatively large reduced volume. The vesicles with relatively small spontaneous curvatures were studied to ensure the stability of oblate vesicles. The vesicles with concave, v=0.80, and convex, v=0.99, shape and also with the shape, which is approximately flat at the poles of the vesicle, v=0.85 were examined. For this range of the reduced volume 0.8<v<0.99 we were able to examine simple vesicle shapes, which did not undergo significant shape transformations with the change of the reduced spontaneous curvature $c_{0}$. We have calculated the adhesion strength $w_{min}$ for which the adhered vesicle has the same energy as the free vesicle for different values of the reduced spontaneous curvature. The value of $w_{min}$ was determined by calculating the reduced free energy *f* for several values of the reduced adhesion strength *w* and reading off $w_{min}$ for the value of the reduced free energy of the free vesicle. It follows from the plots in the first row of Figure 6 that the concave vesicles are stabilized for smaller and smaller values of the adhesion strength with increasing values of the reduced spontaneous curvature $c_{0}$.

Contrary to the concave (v=0.80) vesicles, for the convex vesicles (v=0.99), the adhesion strength $w_{min}$ increases with the increasing reduced spontaneous curvature $c_{0}$. We should stress that the convex vesicle is almost spherical, close to the limiting shape with the reduced volume v=1.0. These features may play a significant role in the process of adhesion. In all the cases, the radius of adhesion $r_{min}$ decreases with the increasing reduced spontaneous curvature within the studied range of $c_{0}$. The changes are very small and can hardly be noticed in the shape profiles of the vesicles. However, the tendencies in the changes induced by the increase of the reduced spontaneous curvature are clearly illustrated.

It is interesting to note that the increase of the spontaneous curvature promotes the adhesion of oblate vesicles to a flat substrate. Smaller adhesion strength $w_{min}$ is needed to obtain stable adhered vesicles. It should be noted that at the same time the radius of adhesion $r_{min}$ is decreasing with increasing spontaneous curvature. Intuitively, one would expect opposite behavior, since it should be favorable for the vesicles with the spontaneous curvature close to zero to adhere to a flat surface with zero mean curvature. When we consider the local mean curvature on a surface of an adhered vesicle we find out that the surface of the membrane attached to a flat substrate is small compared to the remaining surface area of the vesicle, which is characterized by non-zero mean curvature.

We have also investigated the susceptibility of vesicles to adhesion for different shapes of the vesicles. The susceptibility to adhesion may be quantified by the size of the surface area of vesicle’s membrane attached to the substrate. In our case, where we have rotational symmetry, the amount of the vesicle membrane attached to the rigid planar surface can be measured by the the adhesion radius $r_{adh}$.

**Figure 7:**
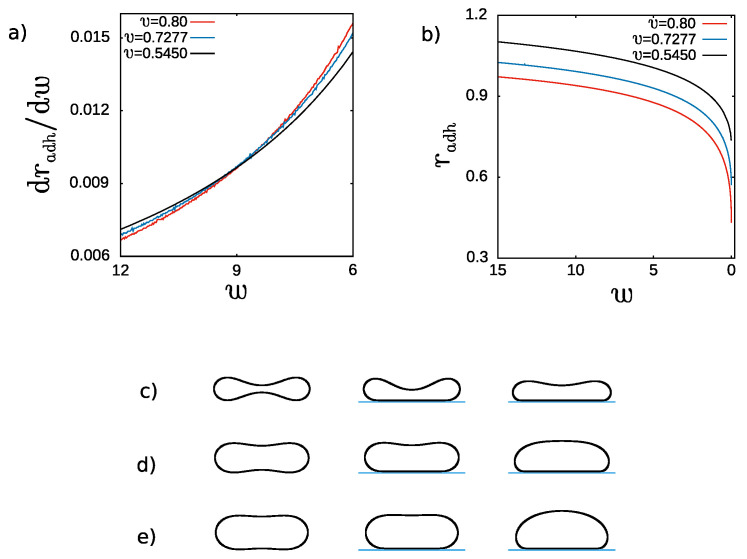
(**a**) The rate of change of the reduced adhesion radius, $dr_{adh}/dw$, and (**b**) the reduced adhesion radius $r_{adh}$ as a function of the reduced adhesion strength *w* for oblate vesicles with three reduced volumes v=0.80;0.7277 and 0.545 and reduced spontaneous curvature $c_{0}=2.4$. The shapes of oblate vesicles for w=0.0;1.0;12.0 in subsequent columns for different values of the reduced volume: (**c**) v=0.545 (**d**) v=0.7277, (**e**) v=0.80.

The rate of change $dr_{adh}/dw$ of the adhesion radius $r_{adh}$ as a function of the adhesion strength *w* for oblate vesicles with different reduced volume v=0.80,0.7277,0.545 and the same spontaneous curvature $c_{0}=2.4$ is presented in Figure 7. As expected for these values of the reduced volume, the rate $dr_{adh}/dw$ decreases monotonically when the adhesion strength is increased for all three values of the reduced volume *v*. We can deduce that it is more and more difficult to attach larger and larger pieces of the vesicle membrane by increasing the adhesion strength by the same value Δw. It should be noted that a linear increase of a radius is equivalent to the increase of the surface area proportional to a radius squared. It can be inferred from Figure 7b that for the same adhesion strength, the largest surface area of adhesion is obtained for the vesicles with smaller reduced volume. The vesicles with smaller reduced volume *v* have more freedom to be deformed since they have smaller inner volume surrounded by the same surface area of a membrane. However, larger adhesion strength is required to obtain stable adhered vesicles with larger reduced volume.

It is interesting to note that for smaller values of the adhesion strength the rate of change of the adhesion radius, $dr_{adh}/dw$, is higher for the vesicles with larger reduced volume, as shown in Figure 7a. For larger adhesion strength this tendency is reversed about w=9. Such behavior might be related to the value of the limiting adhesion radius, which is larger for the vesicles with smaller reduced volume. Thus, it may be expected that the rate of change of the adhesion radius could slow down more for the vesicles with larger reduced volume when the radius is closer and closer to the limiting value.

**Figure 8:**
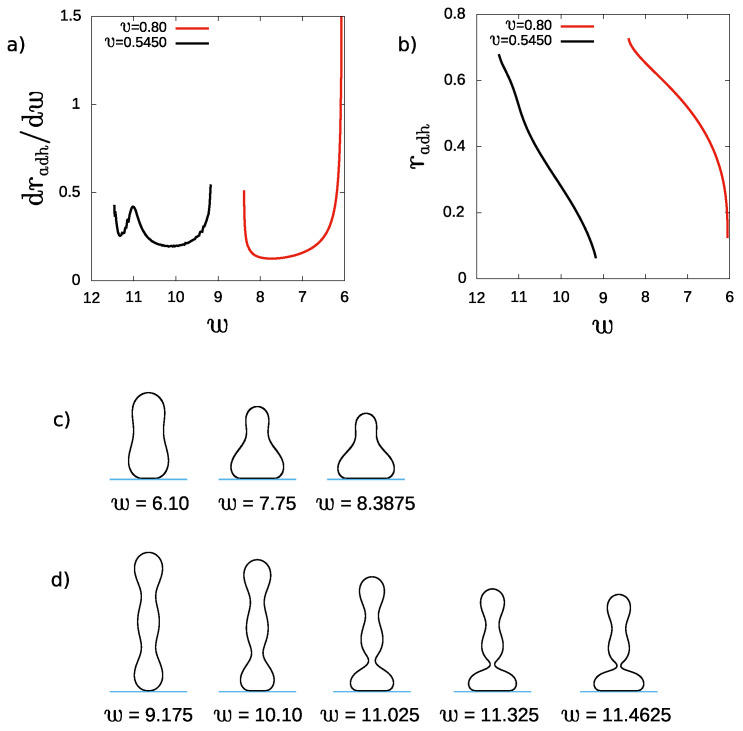
(**a**) The rate of change of the reduced adhesion radius, $dr_{adh}/dw$, and (**b**) the reduced adhesion radius as a function of the reduced adhesion strength, *w* for the vesicles with the reduced volume v=0.80 and 0.545 and reduced spontaneous curvature $c_{0}=2.4$. The shape profiles are plotted in each row for following parameters: (**c**) v=0.80,w=6.10,7.75,8.3875 (**d**) v=0.5450, w=9.175,10.10,11.025,11.325,11.4625.

In Figure 8, we show how the increase of the adhesion strength *w* influences the rate of change of the adhesion radius $r_{adh}$ for prolate vesicles with the spontaneous curvature $c_{0}=2.4$ and the reduced volumes v=0.80 and v=0.545. We would like to investigate how the shape of an adhered vesicle influences its susceptibility to adhesion. We have chosen two vesicles with relatively simple (v=0.80) and complex (v=0.545) shapes. It has to be noted that in the case of adhesion to flat substrate, the vesicles can assume horizontal configurations as the most stable. However, to investigate the role of the shape on the adhesion process we can safely study metastable configurations. Moreover, when a sticker molecule is attached to a pole of a vesicle it is possible to realize the scenario presented by our calculations. The stable adhered prolate vesicles with different reduced volume exist for different ranges of the adhesion strength. Moreover, smaller adhesion strength is sufficient to stabilize adhered prolate vesicles with larger reduced volume.

When prolate vesicles adhere to a flat substrate, the rate of change of the adhesion radius is not monotonous as shown in Figure 8a. For the prolate vesicles with large reduced volume, the rate decreases for smaller values of *w* and increases for larger values of *w*. The shapes of the vesicles at small and large *w* and at the minimum of the rate of change of the adhesion radius $r_{adh}$ are presented in Figure 8c. For larger values of *w* the adhesion leads to transformation of an adhered prolate vesicle to an adhered oblate vesicle. Initially, the vesicle has almost up–down symmetry, but with the increase of the adhesion strength its shape resembles a pear. Finally, the vesicle with a pear-like shape is no longer stable and it is transformed into an adhered oblate one. The rate of change of the adhesion radius increases when the prolate vesicle more and more resembles the oblate one. Thus, before the transformation of the adhered prolate vesicle into the adhered oblate one due to the increase of the adhesion strength, we may expect higher susceptibility to adhesion for the vesicle which is being transformed. Small changes of the adhesion strength may induce large changes of the adhesion radius. Such behavior may be encountered in the vicinity of the shape transformations between different classes of shapes. In such cases we can expect that the adhesion may trigger the transformation of vesicles between two different classes of shapes.

The shape transformations due to adhesion of prolate vesicles with a smaller reduced volume are more complex. The adhesion induces the formation of a narrow neck, which separates the oblate part of the vesicle at the bottom from the upper prolate part, as shown in Figure 8d. The existence of the narrow neck influences the rate of change of the adhesion radius caused by the increase of the adhesion strength, as shown in Figure 8a. With the increasing adhesion strength, the volume of the prolate part becomes smaller and the volume of the oblate part becomes larger. The process ends in a discontinuous transformation of the prolate part into a spherical one. Similarly as in the previous case the rate of change of the adhesion radius increases just before the transformation, as shown in Figure 8a. However, unlike in the previous case this increase is not monotonous. We may attribute this behavior to the existence of the small neck which may stabilize the shape before the transformation. The sequence of shapes which illustrate that process is shown in Figure 8d.

## 3. Summary and Conclusions

The lipid vesicles which adhere to a flat and rigid substrate have been studied. The influence of the adhesion strength on the stability and shape transformations of several types of vesicles have been investigated. It has been shown that the increase of the adhesion strength results in the transformation of stomatocytes to adhered oblate vesicles. The vesicles were characterized by different reduced volume and reduced spontaneous curvature. It has been shown that the increase of the spontaneous curvature promotes adhesion. When a vesicle is attached to the substrate, the local mean curvature at the rim of the attached vesicle increases. An increase of the local mean curvature favors adhesion of the vesicles characterized by high spontaneous curvature, since for these systems the bending energy may be lower due to similar values of the mean and spontaneous curvature.

The minimal strength of adhesion required to stabilize different types of vesicle has been determined. It has been shown that the minimal adhesion strength significantly depends on the reduced volume of a vesicle. The minimal strength of adhesion indicates the transition from a free vesicle to an adhered vesicle. The knowledge of the minimal adhesion strength should be helpful in Atomic Force Microscopy studies where the cantilever touching a cell or a vesicle may cause its detachment.

Lipid vesicles are used as drug carriers. The content of a vesicle is released for example in contact with a malignant tissue. Thus, it is important to know the values of the minimal adhesion strength necessary for the attachment of drug carrying vesicles to different substrates. The calculations performed here for a simple model system may be useful in better understanding the process of adhesion of modern drug carriers in complex biological environment.

In the studied mathematical model it is assumed that the vesicles are tensionless. At large values of the adhesion strength, the shapes of the adhered vesicles are determined by the geometrical constraints. At the limiting values of the adhesion radii, a significant increase of the lateral pressure is expected, indicating possible rupture of the vesicle. The shapes of the vesicles with large spontaneous curvature are complex. During adhesion, the complex structure of the vesicles is altered to obtain flat vesicles’ shapes attached to a large surface area of the substrate. In particular, narrow necks can stabilize complex structure and create barriers for easy attachment of a vesicle to a substrate. In such cases the adhesion may be accompanied by increasing values of the lateral pressure. Such behavior is anticipated based on the calculations of the rate of change of the adhesion radius presented in Figure 8.

The susceptibility to adhesion for different classes of vesicles (oblate, prolate) has been also studied. Even in such a simple model where the vesicles of simple topology adhere to a flat substrate, it is possible to discover many interesting phenomena. We have shown that changing the adhesion strength leads to the formation of a spherical bud or its disappearance.

We propose a mechanism to segregate vesicles based on their shape by creating the adhesion materials with the shape compatible with the vesicle’s geometry. The vesicles which are locally flat can adhere to flat surfaces even with a very small adhesion energy. We may expect that when the surface is locally curved in such a way that it fits to the shape of a vesicle, it will be easy to obtain structures with adhered vesicles. Being able to engineer surfaces with regions of well defined shapes, it will be possible to segregate the collections of different vesicles according to a preferred shape code in the structure of the adhesive material. We can speculate that such segregation governed by adhesion can be used in biotechnological applications to collect nanoparticles like scavengers (leukocytes) do in human body.

In real biological systems such as animal tissues, cells’ membrane can adhere to surfaces, which are not flat and not rigid, for example to the neighboring cells. Considering the relative simplicity of our system, we may anticipate to discover many new phenomena related to adhesion of biological cells to themselves or to rigid objects [1,2,21]. The adhesion of cells may induce novel and very interesting phenomena in large collection of cells in biological tissue or in artificial cell cultures. Moreover, cell–cell and cell-substrate adhesion plays a significant role in highly coordinated motion of touching cells [22]. The loss of the cell–surface adhesion influences the properties and behavior of a cell in collective movement. Thus, it is important to be able to model the shape transformation at the level of a single cell to understand the behavior of large adhering collections of cells. The results of our mathematical modeling of adhesion may be useful in explanation of the behavior of cells cultures confined and grown on a flat substrate [23], as well as in biomedical applications such as protection of the adhesion of platelets to vascular stents [24,25]. 

## References

[1] Ramachandran A., Anderson T.H., Leal L.G., Israelachvili J.N. (2011). Adhesive interactions between vesicles in the strong adhesion limit. Langmuir.

[2] Mareš T., Daniel M., Iglič A., Kralj-Iglič V., Fošnarič M. (2012). Determination of the strength of adhesion between lipid vesicles. Sci. World J..

[3] Fenz S.F., Bihr T., Schmidt D., Merkel R., Seifert U., Sengupta K., Smith A.S. (2017). Membrane fluctuations mediate lateral interaction between cadherin bonds. Nat. Phys..

[4] Evans E. (1980). Analysis of adhesion of large vesicles to surfaces. Biophys. J..

[5] Seifert U., Lipowsky R. (1990). Adhesion of vesicles. Phys. Rev. A.

[6] Seifert U. (1991). Adhesion of vesicles in two dimensions. Phys. Rev. A.

[7] Raval J., Góźdź W.T. (2020). Shape transformations of vesicles induced by their adhesion to flat surfaces. ACS Omega.

[8] Bibissidis N., Betlem K., Cordoyiannis G., von Bonhorst F.P., Goole J., Raval J., Daniel M., Góźdź W., Iglič A., Losada-Pérez P. (2020). Correlation between adhesion strength and phase behaviour in solid-supported lipid membranes. J. Mol. Liq..

[9] Bell G., Dembo M., Bongrand P. (1984). Cell adhesion. Competition between nonspecific repulsion and specific bonding. Biophys. J..

[10] Brochard-Wyart F., de Gennes P.G. (2002). Adhesion induced by mobile binders: Dynamics. Proc. Nat. Acad. Sci. USA.

[11] Steinkühler J., Agudo-Canalejo J., Lipowsky R., Dimova R. (2016). Modulating vesicle adhesion by electric fields. Biophys. J..

[12] Swain P.S., Andelman D. (1999). The influence of substrate structure on membrane adhesion. Langmuir.

[13] Weikl T.R., Asfaw M., Krobath H., Różycki B., Lipowsky R. (2009). Adhesion of membranes via receptor–ligand complexes: Domain formation, binding cooperativity, and active processes. Soft Matter.

[14] Helfrich W. (1973). Elastic properties of lipid bilayers: Theory and possible experiments. Z. Naturforschung C.

[15] Evans E. (1974). Bending resistance and chemically induced moments in membrane bilayers. Biophys. J..

[16] Canham P.B. (1970). The minimum energy of bending as a possible explanation of the biconcave shape of the human red blood cell. J. Theor. Biol..

[17] Döbereiner H.G., Selchow O., Lipowsky R. (1999). Spontaneous curvature of fluid vesicles induced by trans-bilayer sugar asymmetry. Eur. Biophys. J..

[18] Góźdź W.T. (2004). Spontaneous curvature induced shape transformations of tubular polymersomes. Langmuir.

[19] Miao L., Fourcade B., Rao M., Wortis M., Zia R.K.P. (1991). Equilibrium budding and vesiculation in the curvature model of fluid lipid vesicles. Phys. Rev. A.

[20] Góźdź W. (2005). Influence of spontaneous curvature and microtubules on the conformations of lipid vesicles. J. Phys. Chem. B.

[21] Noguchi H. (2019). Detachment of a fluid membrane from a substrate and vesiculation. Soft Matter.

[22] Wang C., Chowdhury S., Driscoll M., Parent C.A., Gupta S.K., Losert W. (2014). The interplay of cell-cell and cell-substrate adhesion in collective cell migration. J. R. Soc. Interface.

[23] Lv J.Q., Chen P.C., Góźdź W.T., Li B. (2020). Mechanical adaptions of collective cells nearby free tissue boundaries. J. Biomech..

[24] Raval J., Gongadze E., Benčina M., Junkar I., Rawat N., Mesarec L., Kralj-Iglič V., Góźdź W., Iglič A. (2021). Mechanical and electrical interaction of biological membranes with nanoparticles and nanostructured surfaces. Membranes.

[25] Benčina M., Rawat N., Lakota K., Sodin-Šemrl S., Iglič A., Junkar I. (2021). Bio-performance of pydrothermally and plasma-treated titanium: The new generation of vascular stents. Int. J. Mol. Sci..

